# The microbiome and resistome of apple fruits alter in the post-harvest period

**DOI:** 10.1186/s40793-022-00402-8

**Published:** 2022-03-07

**Authors:** Birgit Wassermann, Ahmed Abdelfattah, Henry Müller, Lise Korsten, Gabriele Berg

**Affiliations:** 1 Institute of Environmental Biotechnology, Graz University of Technology, Petersgasse 12, 8010 Graz, Austria; 2 Leibniz Institute for Agricultural Engineering and Bioeconomy (ATB), Max-Eyth Allee 100, 14469 Potsdam, Germany; 3 Department of Plant and Soil Sciences, University of Pretoria, Pretoria, Republic of South Africa; 4 DSI-NRF Centre of Excellence in Food Security, Pretoria, Republic of South Africa; 5 Institute for Biochemistry and Biology, University of Postdam, 14476 Potsdam OT Golm, Germany

**Keywords:** *Malus domestica*, Plant, Fruits, Microbiome, Resistome, Antimicrobial resistance, Post-harvest, Food transport

## Abstract

**Background:**

A detailed understanding of antimicrobial resistance trends among all human-related environments is key to combat global health threats. In food science, however, the resistome is still little considered. Here, we studied the apple microbiome and resistome from different cultivars (Royal Gala and Braeburn) and sources (freshly harvested in South Africa and exported apples in Austrian supermarkets) by metagenomic approaches, genome reconstruction and isolate sequencing.

**Results:**

All fruits harbor an indigenous, versatile resistome composed of 132 antimicrobial resistance genes (ARGs) encoding for 19 different antibiotic classes. ARGs are partially of clinical relevance and plasmid-encoded; however, their abundance within the metagenomes is very low (≤ 0.03%). Post-harvest, after intercontinental transport, the apple microbiome and resistome was significantly changed independently of the cultivar. In comparison to fresh apples, the post-harvest microbiome is characterized by higher abundance of *Enterobacteriales,* and a more diversified pool of ARGs, especially associated with multidrug resistance, as well as quinolone, rifampicin, fosfomycin and aminoglycoside resistance. The association of ARGs with metagenome-assembled genomes (MAGs) suggests resistance interconnectivity within the microbiome. Bacterial isolates of the phyla *Gammaproteobacteria*, *Alphaproteobacteria* and *Actinobacteria* served as representatives actively possessing multidrug resistance and ARGs were confirmed by genome sequencing.

**Conclusion:**

Our results revealed intrinsic and potentially acquired antimicrobial resistance in apples and strengthen the argument that all plant microbiomes harbor diverse resistance features. Although the apple resistome appears comparatively inconspicuous, we identified storage and transport as potential risk parameters to distribute AMR globally and highlight the need for surveillance of resistance emergence along complex food chains.

**Supplementary Information:**

The online version contains supplementary material available at 10.1186/s40793-022-00402-8.

## Background

The World Health Organization lists antimicrobial resistance (AMR) among the top 10 global threats to public health and biosecurity, calling for urgent and concerted actions across all sectors as part of the One Health approach [1]. Currently 700,000 people die from antibiotic-resistant bacteria in health care annually and predictions are that numbers will increase to 10 million by 2050 [2]. The development of microbial antibiotic resistance is based on either de-novo mutation or the acquisition of mobile genes from the versatile pool in the environment, which comprises both, naturally evolved antibiotic resistance genes (ARGs) as well as ARGs introduced by anthropogenic practices [3, 4]. Microbial communities are deeply embedded within their host; nevertheless, they represent open and interlinked ecosystems that coevolve, communicate and cross-feed [5–8]. Accordingly, human, animal, and environmental habitats are strongly interconnected, and the effects of applied antibiotics to any of these habitats can extend beyond the site of use [9].

Compared to other human-related environments, food microbiomes, including their resistance potential, are rarely investigated [10]. The microbiome of food is a decisive factor for food quality [11], shelf life [12] and fermentation process [13], and already highlighted as the “missing link” in food safety policies and standards [14]. While health-beneficial microorganisms associated with food have recently been determined to supplement the gut microbiome [15], recent studies showed that raw eaten vegetables and fruits represent an important human–environment interface and can serve as a gateway for environmental AMR to humans [16, 17]. In addition, AMR has been reported of being extraordinary diverse in native plant microbiomes [18, 19] but until now, fresh produce as vector for AMR is less discussed.

Apples are among the most consumed fresh fruits world-wide and serve as important source for health-beneficial metabolites [20], but have also been linked to foodborne outbreaks where contaminations occurred along the processing line [21]. Pre- and postharvest practices affect the microbiome of apple fruits [22–26], while the apple genotype and the geographic location represent important drivers as well [27–29]. However, the apple resistome, nor any other fruit resistome, has been investigated so far and the selection and emergence of AMR during the postharvest period is still unknown. Considering the immense global apple market (129 million tons produced) and trade (9.8 million tons exported) [30], the postharvest period can be an important factor for both, shaping the microbiome and the resistome.

**Fig. 1:**
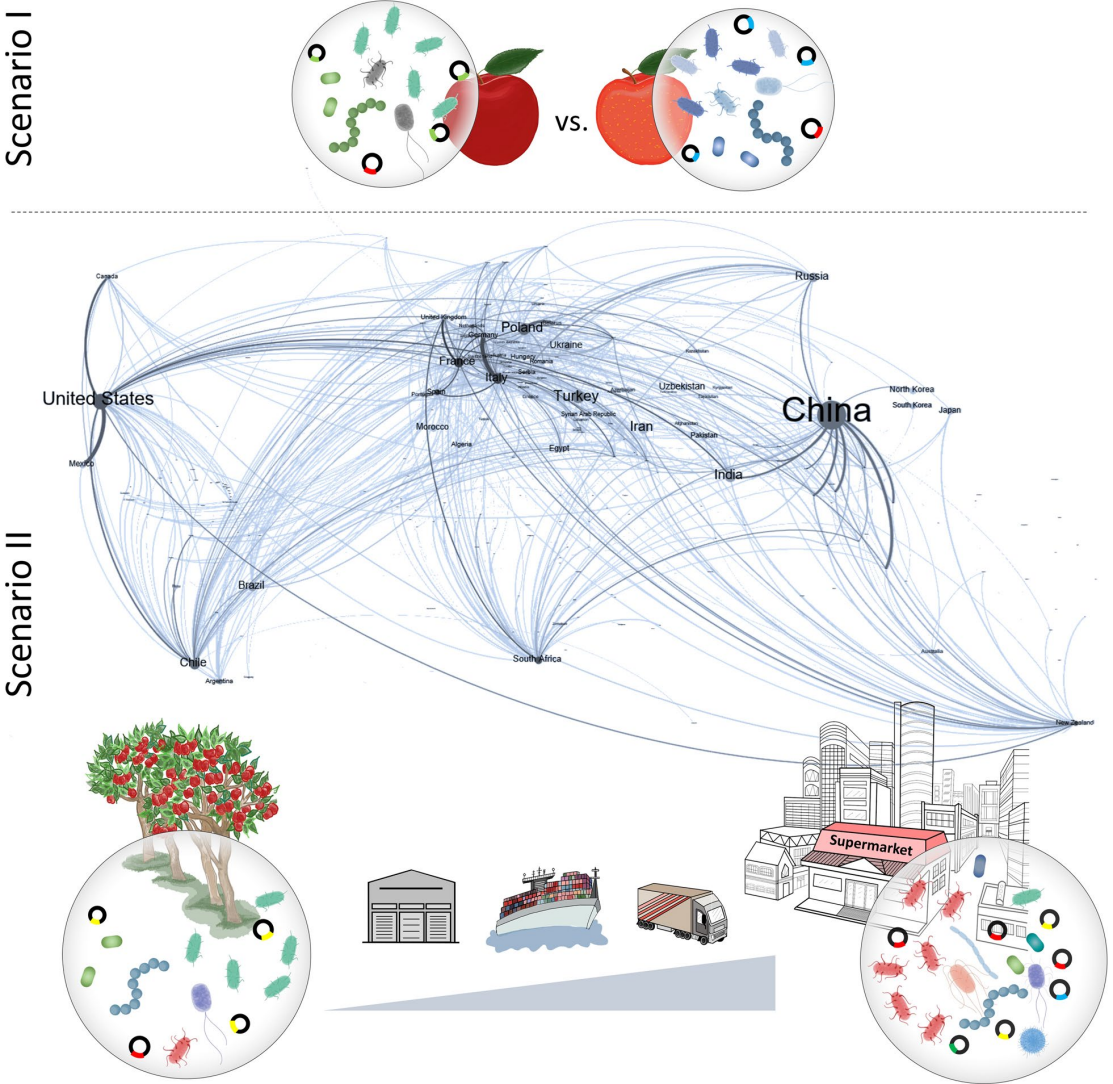
Hypothesized scenarios on the apple fruit microbiome and resistome. In scenario I the apple cultivar (Braeburn versus Royal Gala) drives the microbiome and resistome of the apple fruit. Scenario II suggests an impact of the global apple trade, including storage and transport, on the fruit microbiome and resistome. The map shows the apple production and trade network in 2018. The node size corresponds to apple production according to the data retrieved from [30]. The edge width reflects the export/import quantity between countries according to BACI: International Trade Database at the Product-Level [82]. The network was constructed in Gephi version 0.9.2 [83] using geolayout

To study this objective, we analyzed the microbiome and resistome of apple fruits by applying shotgun metagenomics, 16S rRNA sequencing, quantitative real-time PCR (qPCR) and a cultivation approach including whole genome sequencing (WSG) of multidrug-resistant isolates (Additional file 1: Fig. S1). Apple fruits were studied from two different cultivars (‘Royal Gala’ and ‘Braeburn’) and sources: fresh from the tree and compared them to apples at the end of the intercontinental food system, *i.e.* a supermarket located about 9.000 km away (linear distance: Cape town, South Africa—Graz, Austria). Our hypotheses, comprise two different scenarios: (i) a significant impact of the cultivar, due to the strong filtering effects of the plant genotype on the microbiome [28, 31] and (ii) an impact of transport and storage due to changes in the environment and the metabolic stage of the fruit. In either scenario, the resistome is assumed to reflect the changes occurring in the microbiome structure (Fig. 1).

## Materials and methods

### Sampling procedure and experimental design

Two apple cultivars, Braeburn (BR) and Royal Gala (RG), were selected to investigate the microbiome and resistome of apple fruits and the effect of storage and transport on them. Apple samples were collected at harvest (unprocessed and freshly harvested from trees in two South African orchards; hereinafter referred to as “fresh”) and at the point of sale (stored and transported to Austrian supermarkets; referred to as “stored”). All investigated apples were cultivated in South African orchards under conventional and GlobalG.A.P certified conditions, following industry recommended spray programs. Fresh apples were collected from eight individual trees during harvest time in February 2019 in South African orchards (Braeburn: 33°11′16.1"S 19°15′45.0"E; Royal Gala: 33°11′23.5"S 19°15′12.1"E) and stored fruits were picked randomly from open-layered trays of the same origin in an Austrian supermarket (Graz), at least 30 days after South African harvest time. This reflects the average transport time starting at the day of harvest and includes postharvest processing, transport via ships to Northern European harbors and further truck transport to Austrian supermarkets where it is sold as “in season” South African fruit. All apples were sampled using sterile gloves and sterilized instruments, visually evaluated for consistency in size, shape, and flawlessness, and kept on ice until further processing. We used Illumina MiSeq 16S rRNA amplicon sequences as representatives to estimate bacterial diversity, shotgun metagenomics and genome binning to determine the bacterial composition and AMR profiles, 16S-targeted qPCR for quantification, and a culturomics approach including resistance screening and WGS. The methodological design is outlined in Additional file 1: Fig. S1.

### Bacterial DNA extraction for 16S RNA gene amplicon sequencing and library construction

From each sample category five whole apples were cut into smaller pieces and homogenized in a Stomacher laboratory blender (BagMixer, Interscience, Saint-Nom-la-Bretèche, France) with 20 ml sterile NaCl (0.85%) solution for 3 min. For isolation of apple-associated bacteria and further resistance screening (described below), 1 ml of the suspension was withdrawn. 4 ml of apple suspension was centrifuged (20 min, 16,000×*g*); FastDNA SPIN Kit for Soil (MP Biomedicals, Solon, OH, United States) and a FastPrep Instrument (MP Biomedicals, Illkirch, France) for 30 s at 5.0 m/s was used to extract bacterial genomic DNA from pellets. The primer pair 515f–806r [32] was used for 16S rRNA gene amplification in three technical replicates per sample. Peptide nucleic acid (PNA) clamps were added to PCR mix to block amplification of plant plastid and mitochondrial 16S DNA [33]. PCR was performed in a total volume of 30 μl [5× Taq&Go (MP Biomedicals, Illkirch, France), 1.5 μM PNA mix, 0.25 mM of each primer, PCR-grade water and 1 μl template DNA] applying the following cycling conditions: 95 °C for 5 min, 30 cycles of 96 °C for 1 min, 78 °C for 5 s, 54 °C for 1 min, 74 °C for 60 s and a final elongation at 74 °C for 10 min. Pooled technical replicates were purified by Wizard SV Gel and PCR Clean-Up System (Promega, Madison, WI, United States) and DNA concentrations were measured with Nanodrop 2000 (Thermo Fisher Scientific, Wilmington, DE, United States). All samples were combined in equimolar concentration and sequenced via Illumina MiSeq v2 (250 bp paired end) amplicon sequencing. The same DNA extracts served for qPCR (described below).

### Microbial DNA extraction for shotgun metagenome sequencing

For total microbial DNA extraction, three whole apples from each sample category (BR fresh, RG fresh, BR stored, RG stored) were separately processed, followed by pooling DNA extracts of the three replicates in order to reach sufficient amounts of DNA for subsequent metagenomic shotgun sequencing. For specific enrichment of the microbial cell fraction, a density gradient centrifugation, as previously described [34, 35], was applied. In short, one whole apple was cut in pieces and per 100 mg apple, 500 ml of bacterial cell extraction (BCE) buffer were added and subsequently homogenized with a blender. From this mixture, 100 µl were used for cultivation, as described below. The remaining mixture was filtered through a layer of sterile Mesoft® filters and the filtrate was divided into ten 50 ml tubes. The filtrates were centrifuged (5 min, 10 °C, 500×*g*) and the resulting supernatants were transferred to clean tubes. After an additional centrifugation step (20 min, 10 °C, 5500×*g*), supernatants were discarded, and pellets were resuspended in 50 ml BCE buffer. Suspensions were filtered again through layers of Mesoft® filters and centrifuged (10 min, 10 °C, 10,000×*g*); the resulting pellet was resuspended in 50 ml BCE buffer. Filtration and centrifugation steps were repeated twice. The final filtrates from ten tubes per apple were suspended in 0.5 ml 50 mM Tris HCl (pH 7.5) and pooled. The resulting suspension was overlaid with 4 ml Histodenz™ (Merck, Vienna, Austria) solution (8 g Histodenz dissolved in 10 ml of 50 mM Tris HCl pH 7.5; utilized as alternative to Nycodenz®), and centrifuged (40 min, 10 °C, 10,000×*g*). The bacterial cell fraction, visible as whitish band at the interface of upper and lower phase, was collected and DNA was extracted using FastDNA SPIN Kit for Soil (MP Biomedicals, Solon, OH, United States) and a FastPrep Instrument (MP Biomedicals, Illkirch, France) for 30 s at 5.0 m s^−1^. The three replicates per sample category were combined into one tube, DNA concentrations were measured with Qubit™ 4 Fluorometer (Thermo Fisher Scientific, Wilmington, DE, United States) and the whole DNA extract was sent for metagenomic shotgun sequencing to Vienna BioCenter (Vienna, Austria) using NovaSeq 6000 instrument. DNA was enzymatically fragmented using Westburg NGS DNA Library Prep Kit (Westburg, Leudsen, the Netherlands) before sequencing.

### Quantitative Real-Time PCR (qPCR)

Bacterial 16S rRNA gene copy numbers were quantified via qPCR using the primer pair 515f–927r (10 µM each; [36]). Standard curves for estimation of bacterial abundance were generated using serial dilutions of plasmid DNA containing a full-length copy of *B. subtilis* Sd3-12 16S rRNA gene. The reaction mixes contained 5 µl KAPA SYBR Green, 0.5 µl of each primer, 3 µl PCR-grade water and 1 µl template DNA (diluted 1:10 in PCR grade water). Fluorescence intensity was detected in a Rotor-Gene 6000 real-time rotary analyzer (Corbett Research, Sydney, Australia) applying the following cycling conditions: 95 °C for 5 min, 40 cycles of 95 °C for 20 s, 54 °C for 30 s, 72 °C for 30 s and a final melt curve of 72 to 96 °C. Three individual qPCR runs were conducted for each replicate and intermittently occurring gene copy numbers detected in negative control samples were subtracted from the respective sample.

### Cultivation-dependent resistance screening of bacterial isolates

A 100 µl aliquot of each replicate resulting from tissue homogenization, (described above) was used to isolate cultivable bacteria from apple fruits. Serial dilutions of the suspensions were plated on different media including PDA, R2A, SNA (all from Roth, Germany) and MIS [37]. Plates were incubated at room temperature for four days and subcultured for purification. In total, 160 isolates were picked randomly and screened in triplicates against eight different antibiotics on Müller-Hinton agar plates. Applied concentrations of antibiotics refer to previous publications [17, 19, 38] which followed the guidelines by the Clinical Laboratory Standard Institute: ampicillin: 100 µg ml^−1^, chloramphenicol: 30 µg ml^−1^, erythromycin: 30 µg ml^−1^, gentamycin: 50 µg ml^−1^, penicillin G: 100 µg ml^−1^, rifampicin: 200 µg ml^−1^, tetracycline: 200 µg ml^−1^, vancomycin: 50 µg ml^−1^. Plates were incubated at room temperature for three days and isolates resistant against at least three different antibiotics, thus, considered as multiresistant, were differentiated by BOX-PCR fingerprinting [39] and subsequent 16S rRNA gene Sanger sequencing (LGC Genomics, Berlin, Germany) and NCBI Blast alignment. This way, 12 unique isolates were identified and subjected to whole genome sequencing (WGS).

### Genomic DNA extraction from bacterial isolates and whole genome sequencing

Genomic DNA was extracted from 12 isolates considered as multiresistant using the MasterPure DNA purification kit (Epicenter, WI, USA) and DNA quantity and quality was checked by spectrophotometry (Nanodrop 2000c, Thermo Fisher Scientific, MA, USA) fluorometry (Qubit 4, Thermo Fisher Scientific, MA, USA) and gel electrophoresis. Genomic DNA was sequenced to about 200× coverage using Illumina Novaseq 6000 instrument (150 bp paired-end sequencing; GENEWIZ, Leipzig, Germany).

### Bioinformatics and resistome analysis of the bacterial community of apple fruits

For 16S rRNA gene amplicon analysis, forward and reverse paired end reads were joined in QIIME 1.9.1., imported into QIIME 2 2019.7 and demultiplexed according to QIIME 2 tutorial. Reads were quality-filtered, denoised, chimeric sequences were discarded, and amplicon sequence variants (ASVs) were identified using DADA2 algorithm. In total, 166,460 reads were recovered (7927 mean reads per sample), which were assigned to 1,274 ASVs. Feature classification was performed using a Naïve-Bayes feature classifier trained on Silva132 release [40] and mitochondria and chloroplast reads were discarded. ASV tables were rarefied to an even library size of 2174 prior to alpha and beta diversity calculations, which were assessed running the core diversity script in QIIME 2. Statistics are based on Kruskal–Wallis test for alpha diversity and Analysis of Similarity (ANOSIM) test for beta diversity.

Analyses of the shotgun metagenomic datasets started by removing Illumina adaptors, read truncation to a minimum length of 50 bp and a phred score of 20 in a sliding window of 4 bp using trimmomatic [41]. In order to reduce host-derived sequences in the dataset, forwards and reverse reads were aligned against the reference genome of *Malus domestica,* available at NCBI database (GCF_002114115.1_ASM211411v1_genomic), using Bowtie2 v2.4.1 [42] in very-fast-local alignment mode. After discarding aligned reads, the four metagenomes sequenced produced between 15 and 21 million high quality reads. SAMtools [43] was used to convert Bowtie2 output files and Kaiju v1.7.2 [44] was used for taxonomic classification of sequencing reads. Kaiju output was further used to estimate microbial abundance and richenss, where the latter refers to the total number of differently assigned features. All resistome analyses described in the following were conducted focusing on assembly-based data by using contigs and bins. For particular reference, results of read-based analysis of resistance genes can be looked up in Additional file 1: Table 1. Assembly-based data were generated as follows: paired-end reads were subjected to de novo assembly into contigs using MEGAHIT v1.2.9 [45]. Assembly results are listed in Additional file 1: Table 2. Only contigs with a minimum length of 500 nucleotides (between 400,000 and 600,000 contigs per metagenome) were used for further resistome analyses. Reads were mapped back to assemblies using Bowtie2 v2.4.1 prior to resistance gene annotation with DIAMOND BLASTX (v0.9.29.130) against deepARG v2.0 [46] database. A cutoff of 80% similarity to the reference genes and an e-value of 10^–11^ was set for antibiotic resistance genes to be retained in the dataset. Resistance genes annotated to more than three drug classes were considered as multidrug resistant. To overcome false positive results due to sequencing depth, ARG counts were rarefied using a threshold of 15,851,105, based on the sample with lowest number of reads after removal of host reads. PlasFlow [47] was used to check whether resistance genes, with at least 1000 bp length, are located on either chromosomes or plasmids and RAWGraphs [48] was used to visualize abundance and distribution of plasmid-encoded genes in apple resistomes. Networks of resistance genes were conducted in Cytoscape v3.8.2 [49] and a dendrogram, based on Euclidean distance and average clustering method, was produced in R v4.0.2 with standard function to visualize hierarchical relationship between the resistomes of the four apple samples. CIRCOS Table Viewer v0.63–9 [50] was used for circular representation of ARG relative abundance within the apple samples. Contigs were further binned into draft genomes (metagenome-assembled genomes; MAGs) using MaxBin 2.0 [51] and binning quality was validated with CheckM [52]. Draft genomes with more than 70% completeness and less than 25% contamination were considered for downstream analysis and are listed in Additional file 1: Table 3. Contigs of each genome bin were re-annotated using AmphoraNet [53] and resistance gene annotation was conducted with deepARG database using the same parameters as described above for contigs-based analysis. Relative abundance of each MAG within the respective metagenome was calculated based on the proportion of total bin copy length to the total length of host sequence-filtered reads and a phylogenetic tree was generated based on average nucleotide identity (ANI).

Whole genome bioinformatics and resistance gene annotation started by quality filtering using trimmomatic [41], with the thresholds described above.. Genomes were assembled to scaffolds using SPAdes v.3.15.0 [54] and assembly quality was evaluated with checkM (Additional file 1: Table 4). ANI was applied for taxonomic assignment and construction of the phylogenetic tree. Resistance gene annotation of genomes was conducted as described above for the resistome analysis of shotgun metagenomics datasets.

## Results

### Bacterial composition, diversity, and abundance profiles of fresh and stored apples

We used 16S rRNA gene amplicon sequencing, shotgun metagenomics and qPCR analyses to determine differences in bacterial community composition, diversity, and abundance of apple fruits. Fresh apples at harvest (Br fresh, RG fresh) differed significantly in their community profiles from apples after storage (BR stored, RG stored) (*R* value = 0.5, *p* = 0.01; Additional file 1: Fig. S2A), while no difference was observed when the samples were grouped by the apple cultivar. Bacterial alpha diversity was higher in stored compared to fresh apples. The difference was insignificant for Shannon diversity (Additional file 1: Fig. S2B), but significant for species richness (*p* = 0.02). Correspondingly, using the number of differently assigned features from the metagenomic dataset as hallmark for microbial richness resulted in significant differences as well (BR stored: 29,130 features; RG stored: 27,809; BR fresh: 25,643; RG fresh: 24,442). In contrast, qPCR measurement of total bacterial abundance revealed no difference between apple fruits, amounting between 2.3 × 10^6^ and 4.3 × 10^6^ 16S rRNA gene copy numbers per apple (Additional file 1: Fig. S2C).

**Fig. 2:**
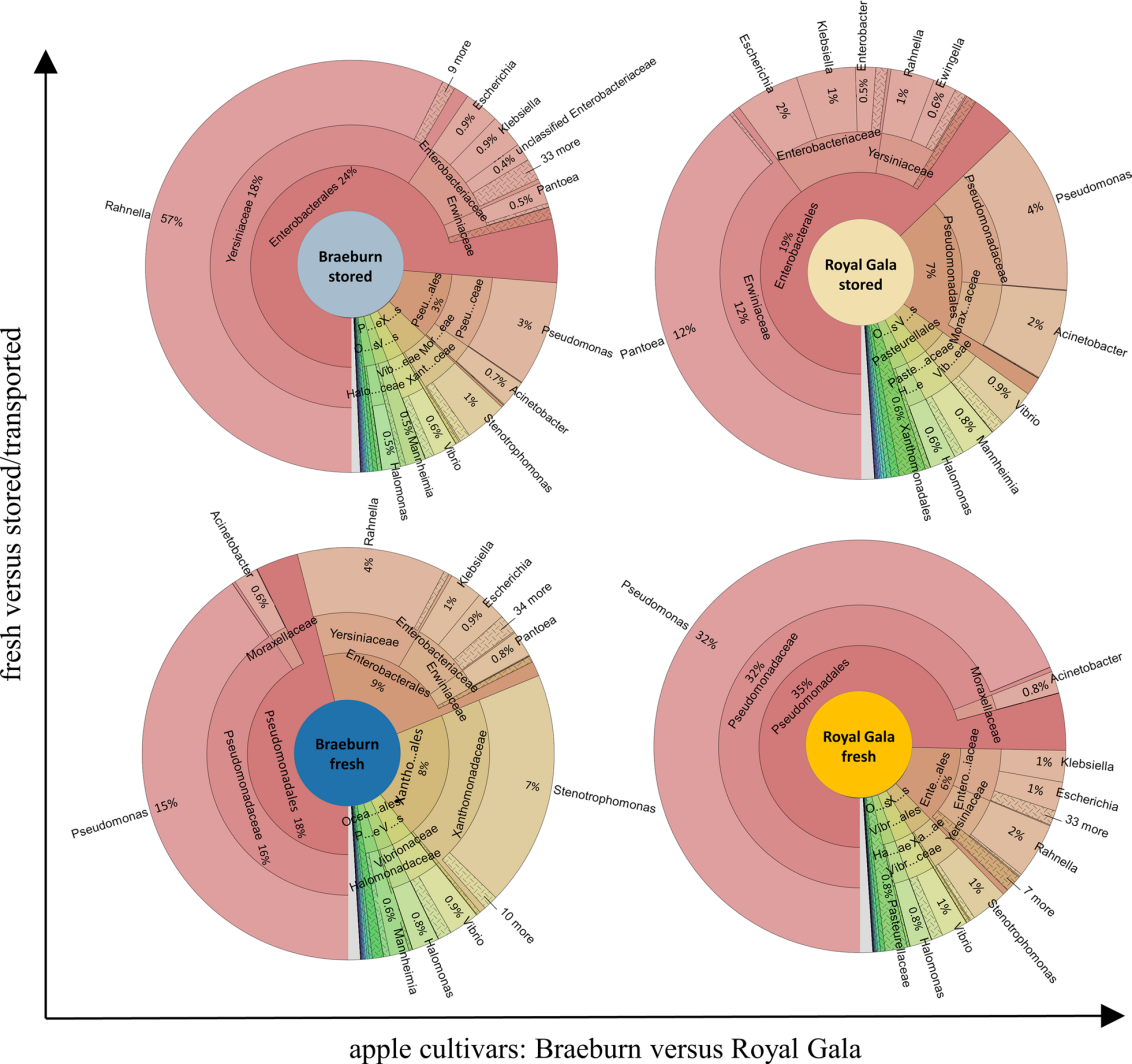
Gammaproteobacterial community profile of apple samples. Multi-level krona plots depict relative abundances of bacterial genera from the class *Gammaproteobacteria* in fresh and stored apples from the cultivars Braeburn and Royal Gala. Please note that percentage values indicate proportion of the respective genus to the whole bacterial community. Bacterial taxonomy was annotated using Kaiju. The entire bacterial composition to order level of each metagenomic sample can be looked up in Additional file 1: Fig. S3

Bacteria was the dominating component of the apple microbiomes, representing 85–87% of the total reads sequenced (Additional file 1: Fig. S3), with *Proteobacteria* covering 57–61% of assigned reads, followed by *Actinobacteria* (9–15%), *Bacteroidetes* (10–13%) and *Firmicutes* (8–13%). *Gammaproteobacteria* was the dominating class in all apples (31–47%), followed by *Alphaproteobacteria* (10–22%), *Actinobacteria* (9–14%), *Bacteroidetes* (10–13%), *Bacilli* (7–11%) and *Betaproteobacteria* (1–2%). The relative abundance of *Gammaproteobacteria* also determined the compositional differences between fresh and stored apples. While fresh apples were highly dominated by *Pseudomonadales* with the genus *Pseudomonas* as main representative (15–32% of all bacterial reads), stored apples showed highest values for *Enterobacteriales* which originated largely from the genera *Rahnella* and *Pantoea* (18–12%) (Fig. 2). Bacterial genera that were shared by all apples with a minimum abundance of 1% in at least one of the samples are provided in Additional file 1: Table 5.

### Antimicrobial resistance genes (ARGs) composition of fresh and stored apples

**Fig. 3:**
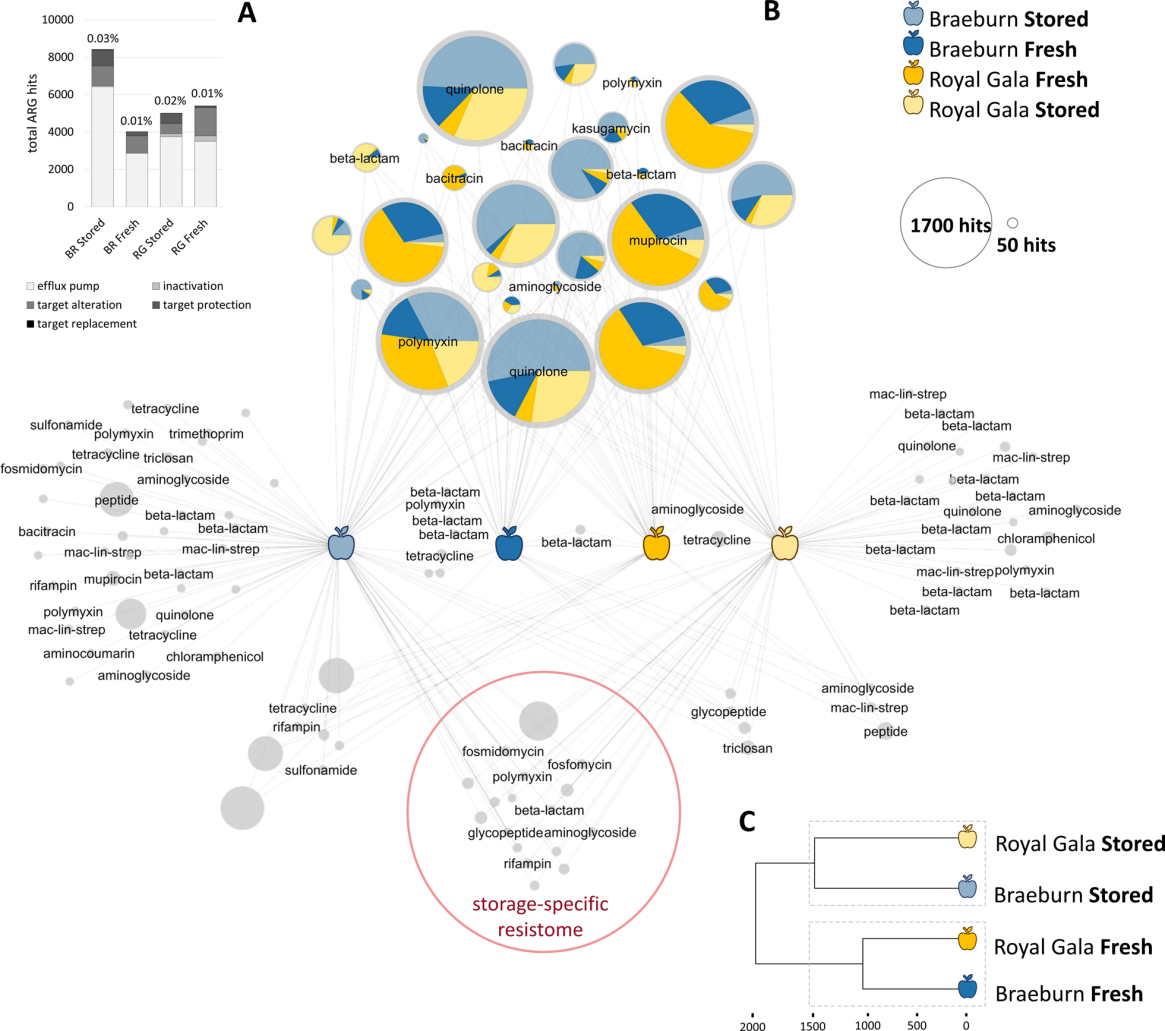
ARG profiles of fresh and stored apple samples. Results are based on deepARG annotations of contigs for resistance genes, target drug classes and resistance mechanisms, including only ARGs with at least 80% similarity to reference ARGs and an e-value of 10^–11^. Data were generated from the rarefied tables. **A** Distribution of resistance mechanisms of annotated ARGs for each apple metagenome. Value on top of each stacked bar denotes for percentage of annotated ARGs to total contigs assigned. Color code for figure panel B and C is depicted in the legend on the upper right: Braeburn: blue; Royal Gala: yellow, both fresh from the tree (dark blue and dark yellow, respectively) and stored/transported (light blue and light yellow, respectively). **B** Network representation of core and specific ARGs in apple metagenomes. Nodes represent different ARGs detected and node labels point to the antibiotic target class, while unlabeled nodes indicate multidrug resistance of the respective ARG. Node size corresponds to absolute abundance of ARGs in the rarefied datasets as indicated in legend on the lower right. Pie charts of nodes shared by all samples, representing the ‘apple core resistome’, indicate fractions detected within each apple metagenome. ARGs that were shared by stored apples but absent in fresh apples are highlighted as the ‘storage-specific resistome’. **C** Dendrogram visualizes connection between different apple samples based on their ARG composition. Calculations were executed in R using Euclidean distance with average clustering method

The resistome of fresh and stored apples was different in terms of total ARGs detected, their relative distribution within samples, the drug classes to which they encode resistance, and the underlying resistance mechanisms (Fig. 3). Regarding the latter, efflux pumps highly prevailed (57% of all ARG hits), while target alteration, target protection, antibiotic inactivation and target replacement, in ascending order, were detected in all samples as well (Fig. 3A). From all assembled contigs, 0.01% to 0.03% were annotated to 132 different ARGs, which code for resistance against 19 different antibiotic classes (Fig. 3B). All apple metagenomes shared 25 high abundant ARGs with a target spectrum of eight antibiotic classes, among which quinolone, polymyxin and mupirocin resistance prevailed, as well as multidrug resistance (presented via pie charts in Fig. 3, B and listed in Additional file 1: Table 6).

ARG diversity was significantly higher in stored/transported apples compared to fresh fruits (p = 0.004; according to independent *t*-test). In addition, stored apples shared 17 ARGs, that were not present in fresh apples, hereinafter referred to as the ‘storage-specific resistome’ (framed red in Fig. 3B and listed in Additional file 1: Table 6). In contrast, fresh apples shared only two low abundant ARGs (one multidrug, one beta-lactam ARG). No cultivar-specific effect was observed for ARG composition, except for three low abundant ARGs shared by Braeburn samples and one ARG specific for Royal Gala apples. Correspondingly, ARG composition within ‘stored’ and ‘fresh’ was more similar than within the cultivars Braeburn and Royal Gala (Fig. 3C).

### Target drug specificity correlates with storage and transport

**Fig. 4:**
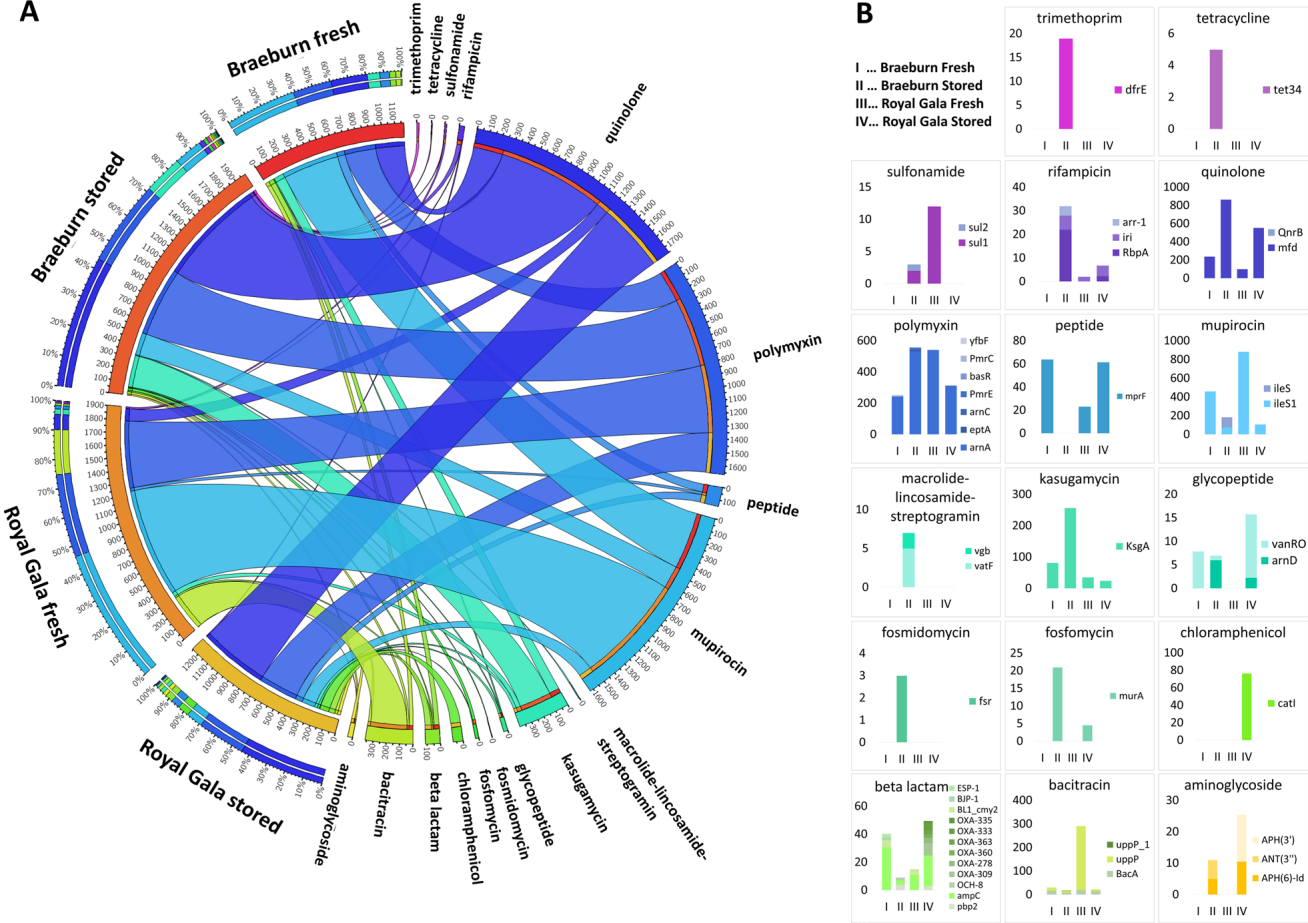
Distribution of drug-specific ARGs in fresh and stored apples. Only ARGs with at least 80% similarity and an e-value of 10^–11^ to reference ARGs are included and ARGs conferring multidrug resistance via efflux pumps, were excluded as they represented 57% of all ARG hits. **A** Circular representation of drug classes based on target-specific resistance gene abundances (right part of the circle) detected in fresh and stored Braeburn and Royal Gala apples (left part of the circle); thickness of ribbons refers to abundance of specific ARGs in the rarefied dataset. Visualization was generated using default settings of Circos software. **B** More detailed classification of the data shown in **A**, where each drug class is visualized in a separate panel. Y-axis of each panel represents total abundance of target-specific ARGs (note the different scaling) within apple samples that are represented on x-axis (I: BR fresh, II: BR stored, III: RG fresh, IV: RG stored). Stacked bars depict ARGs associated to the same antibiotic class within each sample; color-code for ARGs is shown on the right of each panel. *Black arrows* point to antibiotic classes to which resistance gene abundance is either increased or decreased in both stored apples compared to their fresh equivalents

Efflux pumps conferring multidrug resistance highly dominated in all metagenomes. For the purpose of a more in-depth description of target drug-specific ARGs, multidrug-resistant ARGs were excluded from the analysis described in this paragraph. ARGs against quinolones, polymyxin and mupirocin were abundant in all apples (Fig. 4). For stored/transported apples, increased counts for ARGs conferring resistance against quinolone, rifampicin, fosfomycin and aminoglycoside were observed; resistance towards the latter two antibiotics were unique for stored apples. Furthermore, ARGs conferring resistance against trimethoprim, tetracycline, fosmidomycin, chloramphenicol and the combined group of macrolide-lincosamide-streptogramin antibiotics were only detected in either BR stored or RG stored, being absent in fresh apples. In fresh apples, abundances of ARGs acting on mupirocin and bacitracin prevailed. The observed differences were not statistically significant.

### The apple resistome revealed by reconstructed genomes and plasmids

**Fig. 5:**
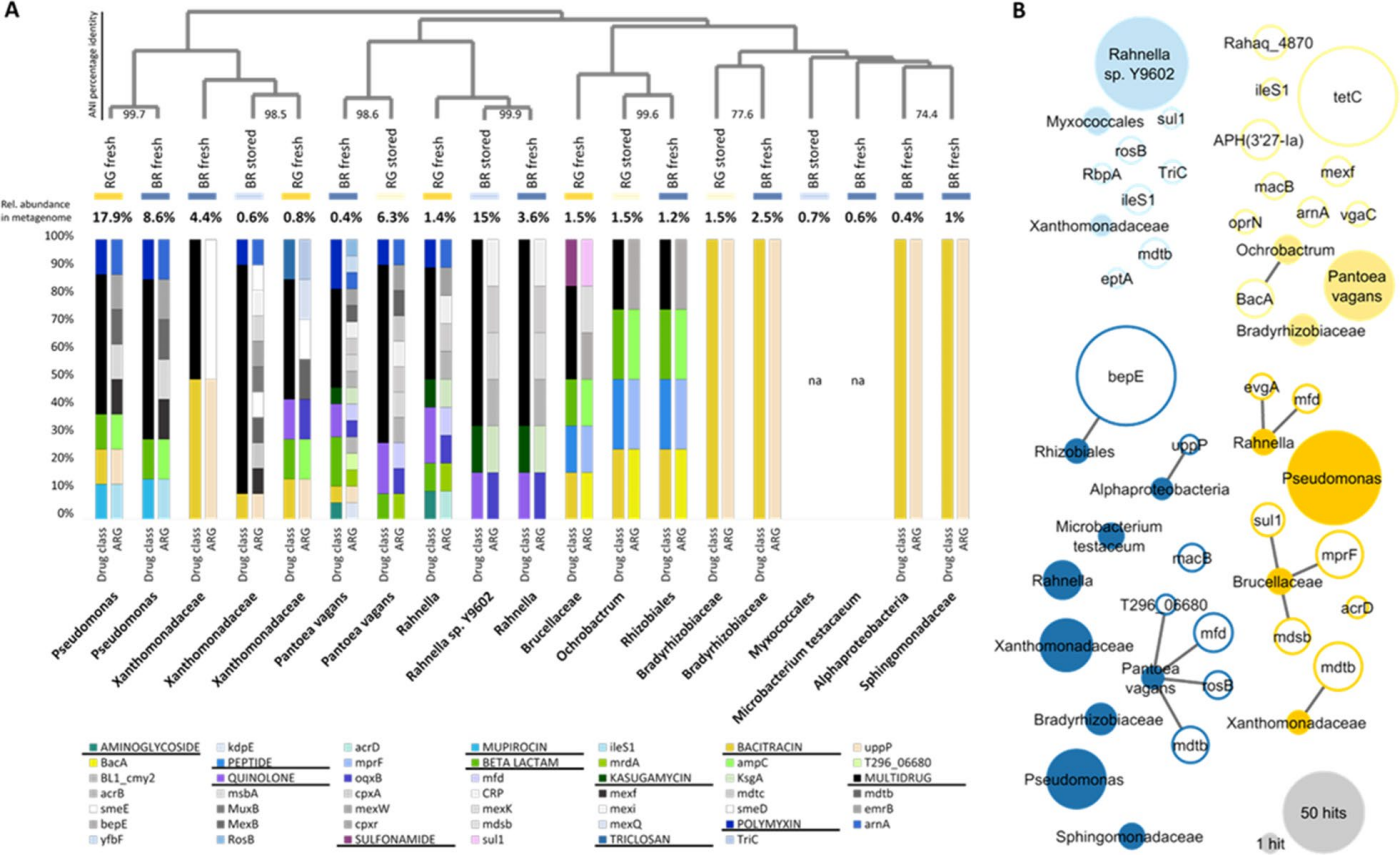
Resistome profiles of reconstructed genomes and plasmids from fresh and stored apples samples. **A** MAGs with sufficient quality are clustered by ANI, values for percentage identity are included for highly similar MAGs, resolved to highest taxonomic levels. Relative abundance of MAGs within the respective metagenome was calculated and is indicated by percentage value on top of each bar. The left bar of each MAG depicts the relative proportion of drug classes to which resistance is conferred by the respective MAG and drug classes are capitalized and underlined in the legend. The right bar represents the ARGs conferring resistance towards the respective drug class. No antibiotic resistant profile was annotated to MAGs assigned to *Myxococcales* (reconstructed from BR stored metagenome) and *M. testacaeum* (BR fresh), as indicated by ‘na’ (not assigned). **B** Resistance networks of MAGs and plasmids. Edge-connected circles indicate potentially transferred resistance genes according to their presence in plasmids and MAGs of the same apple. Size of circles (plasmids: empty circles, MAGs: filled circles) correlate with their total abundance in the respective metagenomes, as shown in the legend on the lower right. Colors of the four groups are defined by apple samples: light blue: BR stored; light yellow: RG stored; dark blue: BR fresh; dark yellow: RG fresh

Assembled contigs were binned into 95 metagenome-assembled genomes (MAGs), of which, 19 MAGs (representing 43.1% of all assembled contigs) had sufficient quality (Additional file 1: Table 3) and were further analyzed in terms of ARG profiles, abundance and similarity (Fig. 5A). High-quality genomes were assigned to *Gammaproteobacteria* (10 MAGs), *Alphaproteobacteria* (7 MAGs, mainly *Rhizobiales*), *Deltaproteobacteria* (1 MAG) and *Actinobacteria* (1 MAG); the latter represents the only MAG of a Gram-positive bacterium. The highest number of genomes was reassembled from BR fresh (9 MAGs), followed by RG fresh (4 MAGs) and BR stored and RG stored each represented by 3 MAGs. The relative abundance of MAGs within their respective metagenomes revealed to be particularly high. For example, *Pseudomonas* with 17.9% abundance in RG fresh, *Pseudomonas* with 8.6% abundance in BR fresh, *P. vagans* in RG stored (6.3%) and *Rahnella* sp. Y9602 in BR stored (15%). Resistance gene annotation for MAGs resulted in 37 different ARGs, with 18 being target-specific to 10 different drug classes and 19 ARGs conferring multidrug resistance. Overall, ARG diversity was significantly higher (*p* = 0.001) for *Gammaproteobacteria* MAGs (e.g. *P. vagans* with 17 different ARGs annotated) than for *Alphaproteobacteria*. No resistance profile was assigned to *Myxococcales* and *M. testacaeum* MAGs. Referring to literature, all ARGs annotated to a MAG in the present work have been previously discovered for representatives of the same bacterial group and all MAGs are, in general, represented by cultivable bacteria.

The fraction of horizontally transferable determinants within the apple resistome was evaluated using the PlasFlow prediction model. From the total of 132 ARGs detected in the dataset, 23 ARGs were assigned to be on plasmids and 72 ARGs to be chromosomally encoded. The number of plasmid-encoded ARGs was similar for all apple metagenomes (BR fresh: 7 ARGs, RG fresh: 7, BR stored: 7, RG stored: 10), with seven being present in more than one apple metagenome, but none being shared by all apples. The comparison of ARGs on MAGs and plasmids from each apple sample separately, revealed potential resistance interconnectivity within the microbiome (Fig. 5B). In total, 13 ARGs could have been transferred between genomes and plasmids; interestingly, between *P. vagans* and *Brucellaceae* MAGs and plasmids, four and three ARGs could have been transferred, respectively. However, higher interconnectivity within fresh apples, as appearing from this analysis, might be biased by the higher number of reassembled genomes from fresh apples.

### The apple resistome revealed by antibiotic susceptibility tests and WGS of isolates

**Fig. 6:**
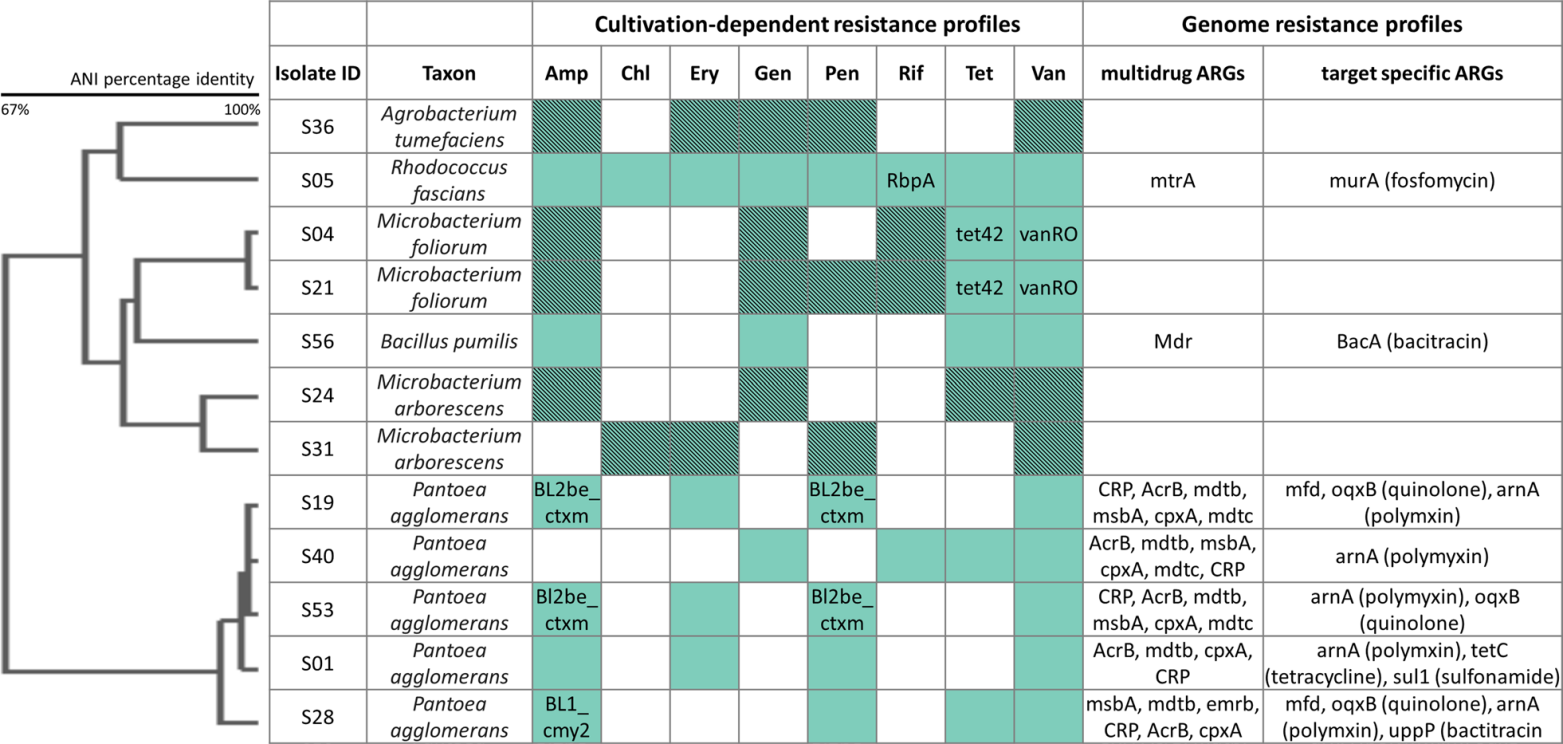
Alignment of cultivation-dependent and genome-annotated resistance profiles of apple isolates. Isolates are clustered and resolved to highest taxonomic levels based on ANI calculation. Colored fields indicate growth of isolates on antibiotic-supplemented agar plates (Amp = ampicillin, Chl = chloramophenicol, Ery = erythromycin, Gen = gentamycin, Pen = penicillin G, Rif = rifampicin, Tet = tetracycline, Van = vancomycin). Green fields mark the corresponding annotation of a potential determinant detected in the sequenced genome (multi resistant genes: ARG ID added in the second last column; target specific ARGs: ARG ID added to respective field). Shaded fields mark resistance profiles which were not confirmed by ARG annotation of genomes. ARG IDs of scaffolds identified as target specific towards antibiotics that were not included in antibiotic susceptibility tests are listed in the last column

From a total of 160 isolates from fresh and stored apple fruits, 36 grew in presence of at least three out of eight antibiotics, thus, in the following considered as multidrug resistant. BOX-PCR fingerprinting and Sanger sequencing resulted in an assignment to twelve different genotypes, dominated by the genera *Pantoea* and *Microbacterium* (Fig. 6). Resistance profiles were observed against all drug classes tested. However, the observed results are not entirely consistent with the metagenomic resistome analysis. In culture collections, resistances against the natural antibiotic vancomycin, followed by ampicillin and penicillin G highly dominated. WGS of isolates followed by ARG annotation confirmed 36 out of 56 of the observed resistances on culture plates. However, the majority of the confirmed resistances can only be explained by multidrug resistant efflux pumps and only few target-specific resistance genes were annotated (*RbpA* for rifampicin resistance of *Rhodococcus fascians*, *tet42* and *vanRO* for tetracycline and vancomycine resistance of *Microbacterium foliourum*, respectively, and *BL2be_ctxm* and *BL1_cmy* for beta-lactam resistance of *Pantoea* isolates). In total, 20 out of the 56 resistance patterns observed on culture plates remained to be unconfirmed by WGS. Especially the profiles of one *Agrobacterium tumefaciens* (resistant against five antibiotics), two *Microbacterium arborscens* and two *M. foliorum* isolates (each resistant against four antibiotics) appear interesting since no resistance genes were annotated to their genomes.

## Discussion

Our study on the microbiome and resistome composition of Braeburn and Royal Gala apples, freshly harvested and after intercontinental fruit trade, provides first insights into the apple resistome available directly to the consumer. Each apple harbors an indigenous, versatile resistome consisting of 132 ARGs, which allows microbiome members to adopt to specific environmental conditions. The postharvest period was identified as a driver of the microbiome as well as the resistome, but not the cultivar. The latter is unexpected since cultivar and plant genotype effects on the plant microbiota are well documented [55–59], even for apples [28, 60–62].

The main compositional difference between the apple microbiomes were detected for *Pseudomonadales* and *Enterobacteriales*, dominating fresh and stored apples, respectively. Both groups are widely distributed in nature and general representatives for the plant microbiome [63]. Thus, the high abundance of *Enterobacteriales* in stored apples might not originate from an external source, but rather from an increase in population size of species native to the apple microbiome. *Enterobacteriales* in general, represent decomposers of plant tissues and constitute characteristic components of a senescent plant. For apple fruits, *Enterobacteriales* abundance has already been reported to increase along with storage time and postharvest treatments [24–26]; the latter might aggravate the general effect of fruit senescence.

The richness of the bacterial community was higher in stored/transported apples, which correlated to higher ARG diversity; especially associated with fosfomycin, aminoglycoside, trimethoprim, tetracycline, fosmidomycin, chloramphenicol and macrolide-lincosamide-streptogramin resistance. Interestingly, most of these drug classes represent (semi-) synthetics that are widely used in clinical environments or livestock treatment including growth promotion [65]. In total, 17 different ARGs were specific for stored apples, but only two ARGs specific for fresh apples, although both cultivars were sampled on the same day, from the same farm, being subjected to the same in-field management. Compared to the most abundant ARGs which were detected in all apples, independent of cultivar or freshness, abundance of these specific ARGs was, however, low. The ARGs shared by all apples were mainly annotated to multi-drug resistant efflux pumps which are known to confer general resistance to a variety of toxic compounds and are suggested as common characteristic for a diversified plant microbiome [19], playing e.g. a role in bacterial colonization and persistence within the host [66]. Resistance features against quinolone, polymyxin and mupirocin were shared by all apples as well and were annotated to MAGs and extracted plasmids. Quinolone and multidrug resistance genes were, however, more abundant and diverse in stored apples. In a recent study, resistomes of unrestricted buildings and controlled built environments were compared, revealing quinolone and multidrug resistance genes to increase with higher levels of confinement [67]. Translating this to agriculture, the postharvest period represents, compared to the field, highly microbial-controlled conditions, which might select for the specific resistant genes.

Our resistome analysis covered also the annotation of ARGs to plasmids, thus, the potential of being transmitted via horizontal gene transfer. Among them, four (*sul1, ileS1, uppP* and *bacA*) should be highlighted due to growing environmental and clinical concerns. Plasmid-borne *sul1* confers resistance towards sulfonamide and synthetic trimethoprim and was detected in fresh and stored apples. Especially in the developing world, the combined usage of trimethoprim/sulfamethoxazole is commonly prescribed as first-line treatment against respiratory and urinary tract infections [68], although rapid spread of resistance among major clinical pathogens is being reported [69–71]. *Sul1* is globally distributed and possess highest environmental fitness, i.e. long-lasting persistence and the ability to proliferate, and is therefore already suggested as indicator gene to assess the antibiotic resistance status of environmental habitats [72]. River sites with strong impact of urban and agricultural activity were reported to correlate with increased trimethoprim/sulfamethoxazole resistance [73]; large-scale intensive agriculture might, thus, correlate also to presence and distribution of *Sul1* in apple. The mupirocin resistance gene *ileS1* was annotated to plasmids of stored/transported apples of both cultivars. Mupirocin, a natural antibiotic produced by *P. fluorescens* [74], is typically used to prevent colonization of methicillin-resistant *Staphylococcus aureus* (MRSA) which can establish critical high-level resistance by carrying *ileS1* [75, 76]. However, for both genes, *sul1* and *ileS1*, no association to MAGs or taxonomic assignment to clinically relevant bacteria and related strains was possible. In contrast, plasmid-borne bacitracin resistance genes *uppP* and *bacA,* were annotated to MAGs of *Alphaproteobacteria* and *Ochrobactrum*, respectively. Bacitracin is widely used in human and veterinary medicine and as animal growth promoter, thus, affecting distinct environments [77]. Across a large-scale metagenomic survey of environmental samples, bacitracin resistance was among the main mechanisms detected in river water and soil [65]. For apples, irrigation systems using river water might be a transmission route for bacitracin resistance, with *Alphaproteobacteria* and *Ochrobactrum*, both general members of soil and rhizosphere communities, representing the vectors.

We isolated bacteria and subjecting them to antibiotic susceptibility tests and confirmed the functional resistance of the apple microbiota against several antibiotics at the applied concentrations. The majority of the resistance profiles were determined on a genetic level as well; however, around one third of observed resistance patterns remains uncertain. This suggests several unknown resistance determinants and gaps in ARG databases, especially for bacterial groups other than *Gammaproteobacteria*. In addition, major resistance profiles observed for isolates (vancomycin, ampicillin and penicillin G) do not entirely correspond to the abundant AMRs assigned to metagenomes. Considering that isolation followed by WGS is state-of-the-art in food safety analyses, our results point out the necessity of combining multi-omics technologies and cultivation assays to obtain a more complete picture of resistomes in a given environment.

Resistance genes can be, in principle, acquired from any source [16], and the apple resistome might be shaped by different aspects. In the field, contamination can occur through irrigation water, organic fertilizers, wild animals, and soil; especially antibiotic usage in animal husbandry or waste water treatment plants is described to co-select for mobile genetic elements that carry multiple resistant genes [72]. The postharvest period, however, represents a critical component which is still less understood. First, handling: apple cultivation and harvest is largely based on manual labor, and in Austrian supermarkets, apples were presented open-layered, assuming further handling. Second, storage time: during storage, host-associated bacteria may evolve towards antibiotic resistance as a natural response to changes of both the host´s physiology, *e.g.* ripening processes and altering metabolic conditions due to changes in the environment i.e. cold storage facilities, containers and refrigerated road transport as well as the metacommunity, including fungi. And third, the variety of postharvest treatments, which mainly target the reduction of microbial loads and diversity [12]. All the mentioned factors, as well as the potential introduction of microbiota from non-agricultural sources, may exert selective pressure which can lead to changes in the community composition and in the intrinsic resistome. Those factors might also result in pleiotropic effects towards distinct traits, including also the coincidental evolution of resistance, even in absence of antibiotics [78]. In return, selective pressure that favors resistance evolution may also alter the composition of the microbiome postharvest. Further studies, monitoring physiochemical parameters throughout the entire processing and transport line, will help to understand the stages that are critical for resistance development in apple microbiomes.

Overall, all fresh and stored apples investigated, revealed a diverse and abundant resistome. Thereby, our study is strengthening the argument that all plant microbiomes harbor intrinsic resistance, as observed under highly heterogeneous trials [18, 19]. Exemplary comparison to recent studies on the *Sphagnum* moss resistome from an undomesticated bog ecosystem [19], and the resistome of leafy-green *Eruca sativa* [17] reveals the apples to contain less associated ARGs and a lower number of resistant isolates. Furthermore, antibiotics are natural products of bacterial secondary metabolism; equally, resistance to antibiotics is a natural and ancient microbial feature, and thus present even in pristine environments that pre-date the anthropogenic influence on resistance dissemination [79, 80]. Finally, the resistome genotype must be distinguished from the resistance phenotype, meaning that the presence of a specific ARG does not encode resistance, inevitably [81]; moreover, some of the genes catalogued as resistant genes in the deepARG database can also be regulators or genes that confer resistance upon their mutation. In view of these facts, the apple resistome does not necessarily call for high alert; however, the observed differences and the potential role of complex food systems and the postharvest period on fruit resistomes should no longer be disregarded. Supported by previous studies on the postharvest apple microbiome [24–26], members of the order *Enterobacteriales,* such as *Pantoea* and *Rahnella,* as well as the ARGs detected specifically in stored apples, should be considered as indicators for reduced fruit and vegetable freshness.

## Conclusion

Overall, the apple microbiome harbors a diverse and versatile resistome. While this is a regular feature of plant-associated communities in general, the current excessive usage of chemicals and antibiotics in agricultural and clinical settings can provoke a shift within produce resistomes. Especially plasmid-encoded ARGs, which were detected in all apple samples, could have an impact. In agreement with the recent suggestion of coordinated *global health* actions to combat world-wide transmission of AMR [9], we suggest the global distribution as well as complex food systems including transport of produce to be considered as a potential risk parameter. We also encourage additional studies which will help to identify resistance genes and microbial carriers of risk for target-oriented monitoring in food safety standards.

## Supplementary Information

**Additional file 1:** List of ARGs following short read-based resistome analysis, quality results of metagenome assembly, binned genomes, and assembled genomes of isolated bacteria, diversity and abundance estimates of 16S rRNA amplicon analysis, bacterial taxonomic composition, and table of ARGs constituting the shared and storage-specific apple resistome.

## Data Availability

The datasets supporting the conclusions of this article are available in the Sequence Read Archive of NCBI. Raw metagenomic reads and 16S rRNA amplicon datasets are available under the BioProject IDs PRJNA734564 and PRJNA734769, respectively. Genome sequences of isolates are accessible under the following BioProject IDs: PRJNA734751, PRJNA734752, PRJNA734754, PRJNA734755, PRJNA734756, PRJNA734758, PRJNA734761, PRJNA734762, PRJNA734763, PRJNA734764, PRJNA734766, PRJNA734768.
