# Supplementary material for: The microbiome and resistome of apple fruits alter in the post-harvest period
Source: Environ Microbiome. 2022 Mar 7;17:10. doi: 10.1186/s40793-022-00402-8 (PMC8900306; doi:10.1186/s40793-022-00402-8)
Supplement: Supplementary file 1 — Additional file 1: List of ARGs following short read-based resistome analysis, quality results of metagenome assembly, binned genomes, and assembled genomes of isolated bacteria, diversity and abundance estimates of 16S rRNA amplicon analysis, bacterial taxonomic composition, and table of ARGs constituting the shared and storage-specific apple resistome. [file 40793_2022_402_MOESM1_ESM.docx]

**Additional file 1**


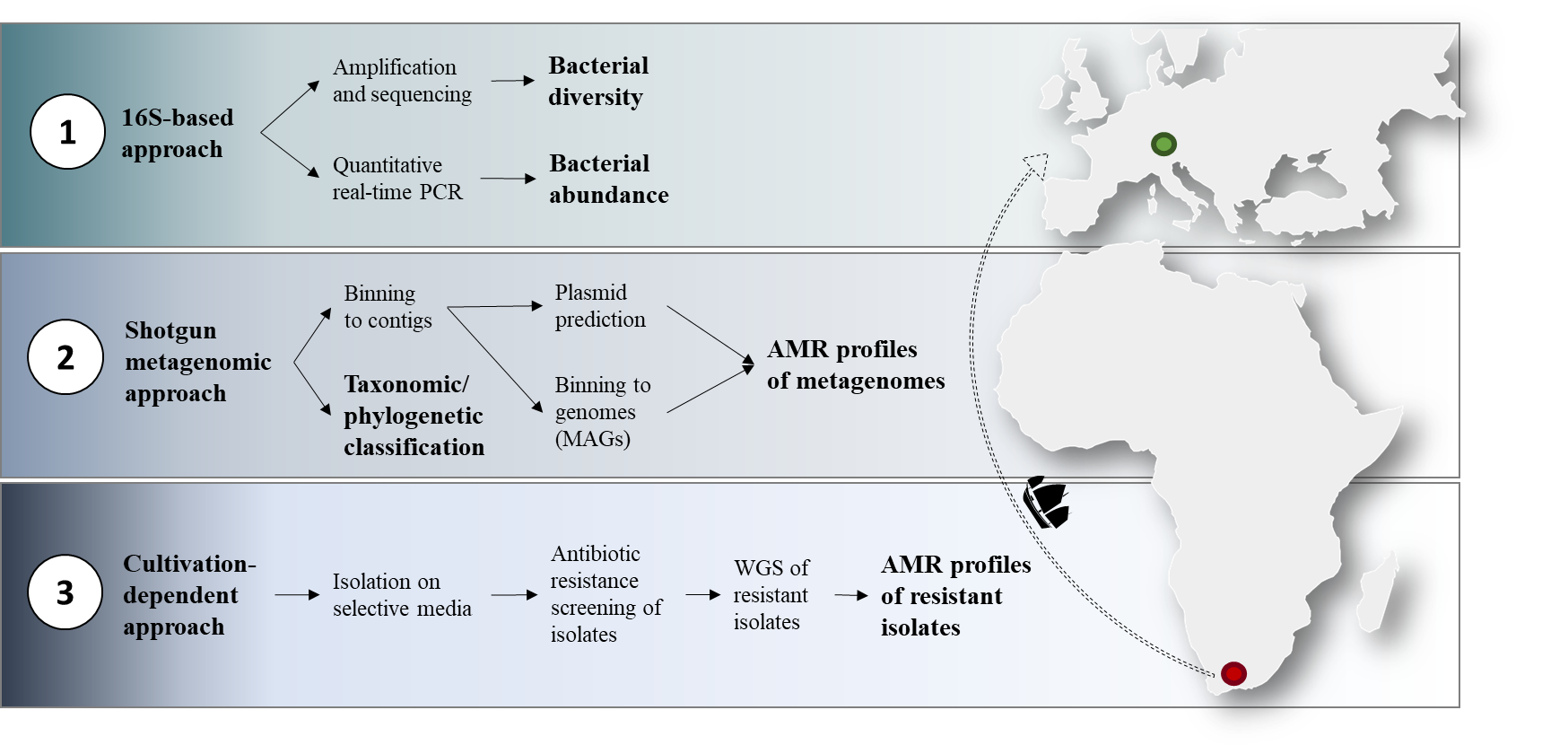
**Supplementary Figure 1:** Overview of applied methods and workflow to analyze the microbiome and resistome of apple fruits from two different cultivars (Braeburn and Royal Gala) and sources (fresh from the tree and after storage and intercontinental transport).

**Supplementary Table 1:** Short read-based resistome analysis

|  | **Braeburn stored** | **Royal Gala stored** | **Braeburn fresh** | **Royal Gala fresh** |
| --- | --- | --- | --- | --- |
| **Drug class** | **Counts of resistance genes** | | | |
| aminoglycoside | 81 | 143 | 29 | 21 |
| bacitracin | 337 | 385 | 310 | 303 |
| beta_lactam | 314 | 525 | 261 | 328 |
| fosfomycin | 3 | 2 | 0 | 0 |
| fosmidomycin | 142 | 31 | 43 | 18 |
| glycopeptide | 6 | 2 | 36 | 65 |
| kasugamycin | 67 | 23 | 26 | 10 |
| macrolide-lincosamide-streptogramin | 16 | 46 | 18 | 4 |
| multidrug | 1863 | 1607 | 1671 | 1788 |
| mupirocin | 67 | 115 | 748 | 1297 |
| polymyxin | 954 | 1156 | 787 | 1063 |
| quinolone | 802 | 701 | 270 | 109 |
| rifampin | 3 | 8 | 6 | 3 |
| sulfonamide | 3 | 19 | 5 | 5 |
| tetracycline | 10 | 179 | 7 | 13 |
| triclosan | 3 | 8 | 13 | 13 |
| trimethoprim | 0 | 2 | 0 | 0 |
| **Total** | **4671** | **5128** | **4257** | **5045** |

**Supplementary Table 2**: Assembly results of apple metagenomes.

|  | **Braeburn fresh** | **Royal Gala fresh** | **Braeburn stored** | **Royal Gala stored** |
| --- | --- | --- | --- | --- |
| **# number of contigs** | 1483942 | 1458819 | 2031535 | 2072091 |
| **# total contigs length** | 1027121064 | 1020881298 | 1266555057 | 1241053195 |
| **# mean contig size** | 692,16 | 699,8 | 623,45 | 598,94 |
| **# contig size first quartile** | 283 | 286 | 315 | 276 |
| **# median contig size** | 352 | 373 | 379 | 337 |
| **# contig size third quartile** | 526 | 605 | 530 | 475 |
| **# longest contig** | 859584 | 361703 | 859491 | 395011 |
| **# shortest contig** | 200 | 200 | 200 | 200 |
| **# contigs > 500 nt*** | 396629 (26.73 %) | 458831 (31.45 %) | 555329 (27.34 %) | 475832 (22.96 %) |
| **# contigs > 1K nt** | 173181 (11.67 %) | 203072 (13.92 %) | 206949 (10.19 %) | 188404 (9.09 %) |
| **# contigs > 10K nt** | 6249 (0.42 %) | 3829 (0.26 %) | 3413 (0.17 %) | 6538 (0.32 %) |
| **# contigs > 100K nt** | 68 (0.00 %) | 33 (0.00 %) | 19 (0.00 %) | 44 (0.00 %) |
| **# contigs > 1M nt** | 0 (0.00 %) | 0 (0.00 %) | 0 (0.00 %) | 0 (0.00 %) |
| **# N50** | 1079 | 1040 | 693 | 737 |
| **# L50** | 159218 | 193919 | 340259 | 276931 |
| **# N80** | 352 | 368 | 356 | 324 |
| **# L80** | 744903 | 747979 | 1174382 | 1130450 |

*Contigs with a minimum length of 500 nt were used for downstream analyses

**Supplementary Table 3**: Summary of binned genomes from apple metagenomics dataset

| **Sample** | **bin No.** | **Marker lineage CheckM** | **Taxonomy AMPHORA2** | **# genomes** | **# markers** | **# marker sets** | **0** | **1** | **2** | **3** | **4** | **5+** | **Completeness** | **Contamination** | **Strain heterogeneity** | **binID** |
| --- | --- | --- | --- | --- | --- | --- | --- | --- | --- | --- | --- | --- | --- | --- | --- | --- |
| Braeburn fresh | 1 | k__Bacteria | Rhizobiales | 5449 | 104 | 58 | 0 | 82 | 18 | 4 | 0 | 0 | 100 | 14,03 | 6,67 | 5,011 |
|  | 2 | f__Enterobacteriaceae | Rahnella | 223 | 875 | 305 | 0 | 857 | 18 | 0 | 0 | 0 | 100 | 2,49 | 16,67 | 5,005 |
|  | 3 | k__Bacteria | Pseudomonas | 5449 | 103 | 57 | 0 | 83 | 18 | 2 | 0 | 0 | 100 | 12,28 | 4,17 | 5,003 |
|  | 4 | f__Xanthomonadaceae | Xanthomonadaceae | 55 | 659 | 290 | 21 | 634 | 4 | 0 | 0 | 0 | 99,57 | 0,51 | 0 | 5,004 |
|  | 5 | o__Rhizobiales | Bradyrhizobiaceae | 107 | 485 | 316 | 3 | 466 | 16 | 0 | 0 | 0 | 99,26 | 3,49 | 12,5 | 5,006 |
|  | 6 | o__Actinomycetales | Microbacterium testaceum | 69 | 399 | 197 | 3 | 325 | 67 | 4 | 0 | 0 | 98,92 | 15,97 | 16,46 | 5,009 |
|  | 7 | o__Sphingomonadales | Sphingomonadaceae | 26 | 569 | 293 | 17 | 524 | 26 | 2 | 0 | 0 | 97,97 | 5,48 | 15,62 | 5,01 |
|  | 8 | f__Enterobacteriaceae | Pantoea vagans | 223 | 875 | 305 | 132 | 703 | 38 | 2 | 0 | 0 | 84,24 | 6,25 | 25 | 5,015 |
|  | 9 | o__Rhizobiales | Alphaproteobacteria | 92 | 481 | 319 | 108 | 281 | 78 | 12 | 2 | 0 | 80,41 | 23,42 | 1,59 | 5,018 |
| Royal Gala fresh | 10 | g__Pseudomonas | Pseudomonas | 78 | 1044 | 368 | 2 | 1035 | 7 | 0 | 0 | 0 | 99,86 | 0,63 | 14,29 | 6,002 |
|  | 11 | f__Xanthomonadaceae | Xanthomonadaceae | 55 | 659 | 290 | 38 | 560 | 59 | 2 | 0 | 0 | 96,69 | 9,92 | 3,08 | 6,009 |
|  | 12 | k__Bacteria | Brucellaceae | 5449 | 104 | 58 | 4 | 71 | 29 | 0 | 0 | 0 | 94,83 | 18,35 | 0 | 6,005 |
|  | 13 | f__Enterobacteriaceae | Rahnella | 223 | 875 | 305 | 87 | 605 | 179 | 4 | 0 | 0 | 92,03 | 24,27 | 17,28 | 6,006 |
| Braeburn stored | 14 | f__Enterobacteriaceae | Rahnella sp. Y9602 | 223 | 875 | 305 | 0 | 865 | 10 | 0 | 0 | 0 | 100 | 0,73 | 10 | 3,003 |
|  | 15 | c__Deltaproteobacteria | Myxococcales | 83 | 247 | 155 | 89 | 155 | 3 | 0 | 0 | 0 | 70,18 | 1,61 | 33,33 | 3,005 |
|  | 16 | f__Xanthomonadaceae | Xanthomonadaceae | 55 | 659 | 290 | 167 | 386 | 87 | 16 | 2 | 1 | 75,55 | 22,08 | 4,46 | 3,016 |
| Royal Gala stored | 17 | f__Enterobacteriaceae | Pantoea vagans | 223 | 875 | 305 | 1 | 860 | 14 | 0 | 0 | 0 | 99,84 | 1,25 | 14,29 | 4,002 |
|  | 18 | c__Alphaproteobacteria | Ochrobactrum | 564 | 349 | 230 | 6 | 320 | 23 | 0 | 0 | 0 | 98,26 | 5,08 | 4,35 | 4,005 |
|  | 19 | o__Rhizobiales | Bradyrhizobiaceae | 107 | 484 | 316 | 13 | 455 | 15 | 1 | 0 | 0 | 97,68 | 3,23 | 16,67 | 4,004 |

| **bin No.** | **Marker lineage** | **# genomes** | **# markers** | **# marker sets** | **0** | **1** | **2** | **3** | **4** | **5+** | **Completeness** | **Contamination** | **Strain heterogeneity** | **binID** |
| --- | --- | --- | --- | --- | --- | --- | --- | --- | --- | --- | --- | --- | --- | --- |
| 1 | f__Enterobacteriaceae (UID5054) | 223 | 875 | 305 | 0 | 872 | 3 | 0 | 0 | 0 | 100 | 0,36 | 0 | S01_scaffoldsMinLen1T |
| 4 | o__Actinomycetales (UID1593) | 69 | 400 | 198 | 1 | 398 | 1 | 0 | 0 | 0 | 99,49 | 0,51 | 0 | S04_scaffoldsMinLen1T |
| 5 | o__Actinomycetales (UID1815) | 120 | 574 | 266 | 1 | 569 | 3 | 1 | 0 | 0 | 99,94 | 1,69 | 0 | S05_scaffoldsMinLen1T |
| 19 | f__Enterobacteriaceae (UID5054) | 223 | 875 | 305 | 0 | 871 | 4 | 0 | 0 | 0 | 100 | 0,41 | 0 | S19_scaffoldsMinLen1T |
| 21 | o__Actinomycetales (UID1593) | 69 | 400 | 198 | 3 | 397 | 0 | 0 | 0 | 0 | 98,99 | 0 | 0 | S21_scaffoldsMinLen1T |
| 24 | o__Actinomycetales (UID1593) | 69 | 400 | 198 | 2 | 393 | 4 | 1 | 0 | 0 | 98,99 | 1,39 | 0 | S24_contigsMinCov150minLen1T |
| 28 | f__Enterobacteriaceae (UID5054) | 223 | 875 | 305 | 1 | 872 | 2 | 0 | 0 | 0 | 99,67 | 0,25 | 0 | S28_scaffoldsMinCov100MinLen1T |
| 31 | o__Actinomycetales (UID1593) | 69 | 400 | 198 | 3 | 392 | 4 | 1 | 0 | 0 | 98,74 | 1,39 | 0 | S31_scaffoldsMinCov150MinLen1T |
| 36 | f__Rhizobiaceae (UID3564) | 78 | 840 | 354 | 17 | 811 | 12 | 0 | 0 | 0 | 97,17 | 1,59 | 0 | S36_scaffoldsMinCov100MinLen500 |
| 40 | f__Enterobacteriaceae (UID5054) | 223 | 875 | 305 | 0 | 873 | 2 | 0 | 0 | 0 | 100 | 0,19 | 0 | S40_scaffoldsMinCov100MinLen500 |
| 53 | f__Enterobacteriaceae (UID5054) | 223 | 875 | 305 | 0 | 872 | 3 | 0 | 0 | 0 | 100 | 0,36 | 0 | S53_scaffoldsMinLen1T |
| 56 | g__Bacillus (UID864) | 93 | 711 | 241 | 1 | 710 | 0 | 0 | 0 | 0 | 99,59 | 0 | 0 | S56_scaffoldsMinLen500 |

**Supplementary Table 4:** Quality results of assembled genomes of apple isolated bacteria


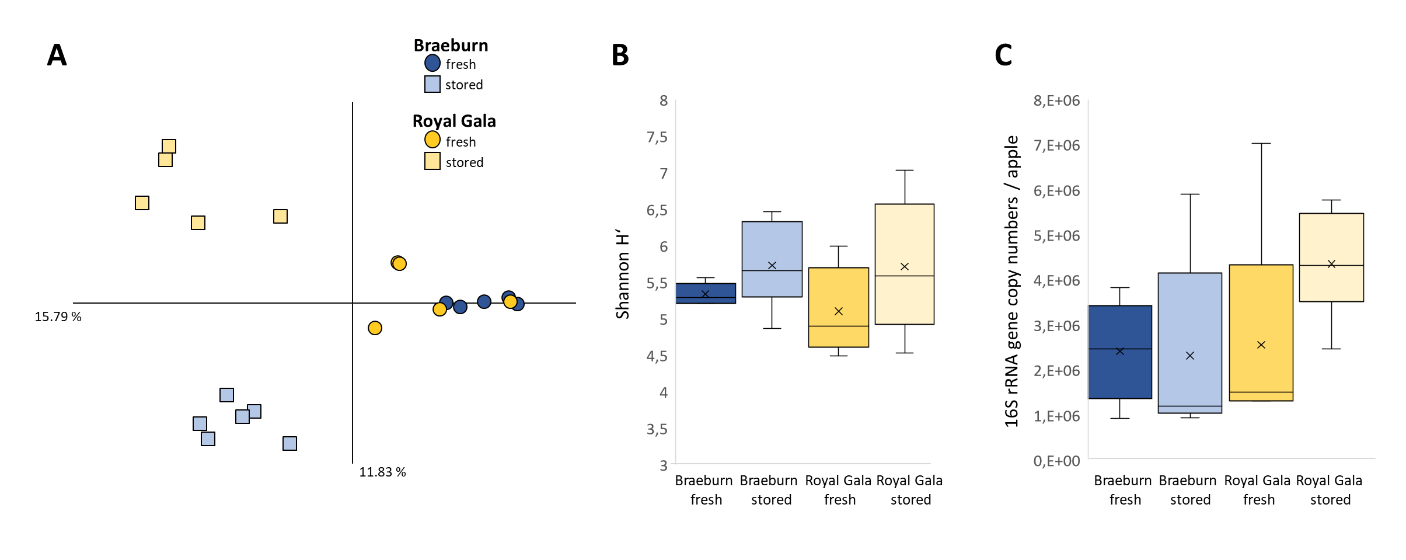


**Supplementary Figure 2**: Bacterial diversity and abundance estimates of fresh and stored/transported apple fruits from two cultivars. (A) PCoA plot visualizing Bray-Curtis distance matrix and (B) Shannon H’ index estimates for alpha diversity are based on 16S rRNA gene amplicon analysis. (C) shows 16S rRNA gene copy numbers per one apple, determined via quantitative real-time PCR.

**
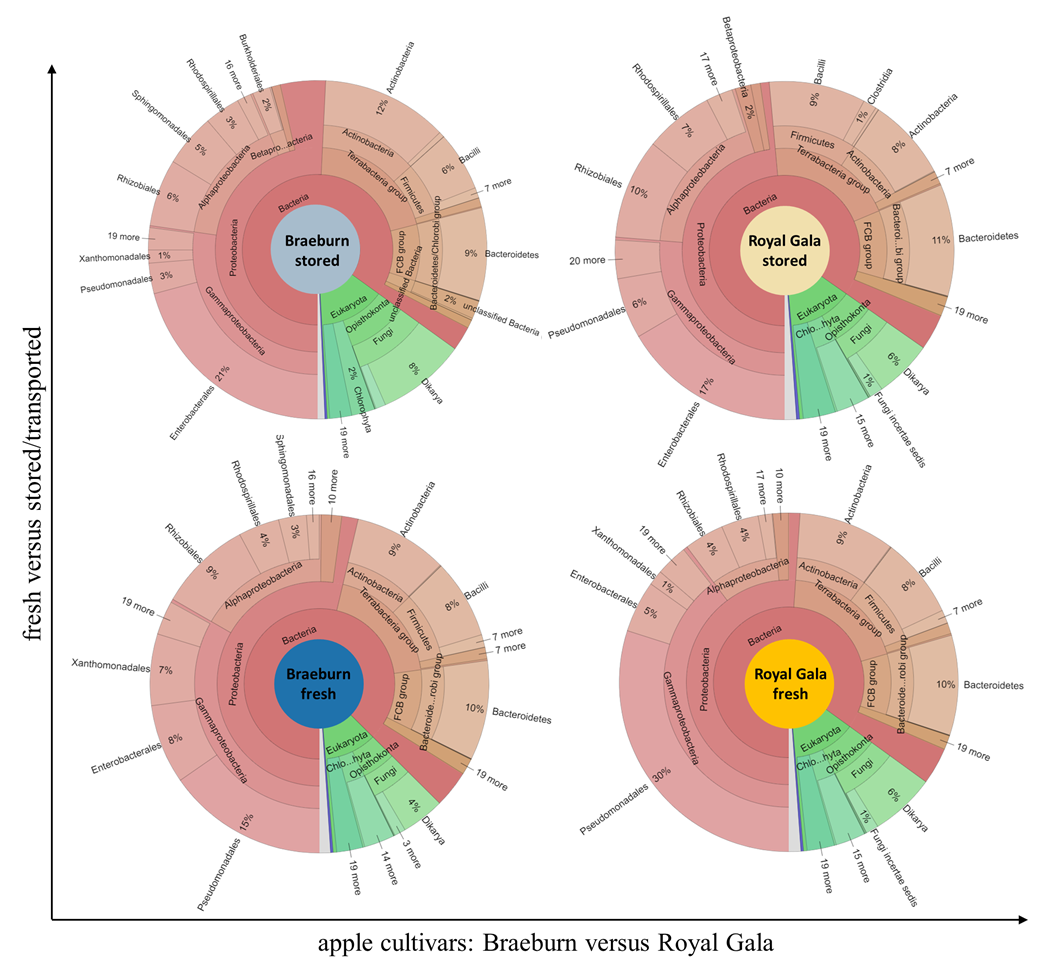
**

**Supplementary Figure 3:** Krona charts visualizing the overall microbial composition of fresh and stored apples from both cultivars. Taxonomy is resolved up to order level and assignment was done using metagenome classifier Kaiju.

**Supplementary Table 5:** Relative abundance of the 18 most abundant bacterial core genera.

|  | **Braeburn** | | **Royal Gala** | |
| --- | --- | --- | --- | --- |
|  | **fresh (%)** | **stored (%)** | **fresh (%)** | **stored (%)** |
| *Pseudomonas* | 15 | 3 | 32 | 4 |
| *Rahnella* | 4 | 18 | 2 | 1 |
| *Acetobacter* | 4 | 3 | 3 | 6 |
| *Frankia* | 4 | 2 | 4 | 4 |
| *Solibacillus* | 3 | 3 | 3 | 5 |
| *Pantoea* | 0,8 | 0,5 | 0,4 | 12 |
| *Staphylococcus* | 3 | 2 | 3 | 3 |
| *Stenotrophomonas* | 7 | 1 | 1 | 0,6 |
| *Sphingomonas* | 3 | 4 | 0,5 | 0,5 |
| *Bradyrhizobium* | 3 | 0,9 | 0,8 | 2 |
| *Microbacterium* | 2 | 0,5 | 2 | 0,4 |
| *Paenanthrobacter* | 2 | 0,9 | 1 | 1 |
| *Escherichia* | 0,9 | 0,9 | 1 | 2 |
| *Methylobacterium* | 0,4 | 3 | 0,07 | 0,8 |
| *Acinetobacter* | 0,7 | 0,7 | 0,8 | 2 |
| *Ochrobactrum* | 1 | 0,08 | 0,9 | 2 |
| *Klebsiella* | 1 | 0,9 | 1 | 1 |
| *Bacillus* | 0,6 | 0,6 | 0,7 | 1 |

**Supplementary Table 6**: ARGs constituting the ‘shared apple resistome’ and the ‘storage-specific resistome’

| **Shared apple resistome** | | | | | | | |
| --- | --- | --- | --- | --- | --- | --- | --- |
| **ARG** | **resistance mechanism** | **target drug class** | **BR fresh** | **RG fresh** | **BR**  **stored** | **RG**  **stored** | **% to annotated ARGs** |
| mfd | target protection | quinolone | 237 | 99 | 862 | 551 | 7,65 |
| oqxB | efflux pump | quinolone | 242 | 93 | 919 | 470 | 7,54 |
| arnA | target alteration | polymyxin | 244 | 541 | 531 | 306 | 7,1 |
| ileS1 | target alteration | mupirocin | 458 | 882 | 75 | 106 | 6,65 |
| mexK | efflux pump | multidrug | 452 | 871 | 85 | 48 | 6,37 |
| MexB | efflux pump | multidrug | 435 | 894 | 54 | 52 | 6,28 |
| MexF | efflux pump | multidrug | 403 | 834 | 46 | 25 | 5,72 |
| acrB | efflux pump | multidrug | 36 | 50 | 789 | 413 | 5,64 |
| msbA | efflux pump | multidrug | 123 | 33 | 516 | 297 | 4,24 |
| cpxA | efflux pump | multidrug | 133 | 55 | 474 | 274 | 4,1 |
| tolC | efflux pump | multidrug | 123 | 48 | 493 | 31 | 3,04 |
| CRP | efflux pump | multidrug | 79 | 35 | 306 | 165 | 2,56 |
| mdtb | efflux pump | multidrug | 33 | 24 | 68 | 397 | 2,28 |
| cpxr | efflux pump | multidrug | 141 | 263 | 18 | 28 | 1,97 |
| KsgA | target alteration | kasugamycin | 81 | 35 | 256 | 24 | 1,73 |
| emrB | efflux pump | multidrug | 25 | 49 | 7 | 279 | 1,58 |
| mrdA | efflux pump | beta_lactam | 27 | 7 | 12 | 312 | 1,57 |
| uppP | inactivation | bacitracin | 14 | 271 | 7 | 7 | 1,31 |
| H-NS | efflux pump | multidrug | 32 | 7 | 167 | 13 | 0,96 |
| bepE | efflux pump | multidrug | 65 | 38 | 3 | 57 | 0,71 |
| ampC | inactivation | beta_lactam | 31 | 11 | 3 | 22 | 0,29 |
| BacA | target alteration | bacitracin | 17 | 20 | 8 | 16 | 0,27 |
| rosB | efflux pump | polymyxin | 8 | 3 | 12 | 32 | 0,24 |
| ceoB | efflux pump | multidrug | 3 | 2 | 31 | 1 | 0,16 |
| acrD | efflux pump | aminoglycoside | 4 | 16 | 12 | 3 | 0,15 |
| **Storage-specific apple resistome** | | | | | | | |
| **ARG** | **resistance mechanism** | **target drug class** | **BR fresh** | **RG fresh** | **BR**  **stored** | **RG**  **stored** | **% to annotated ARGs** |
| mdfA | efflux pump | multidrug | 0 | 0 | 472 | 7 | 2,1 |
| mexW | efflux pump | multidrug | 0 | 0 | 32 | 40 | 0,32 |
| arpB | efflux pump | multidrug | 0 | 0 | 34 | 34 | 0,3 |
| MuxB | efflux pump | multidrug | 0 | 0 | 47 | 4 | 0,22 |
| mtrA | efflux pump | multidrug | 0 | 0 | 39 | 4 | 0,19 |
| arpC | efflux pump | multidrug | 0 | 0 | 20 | 9 | 0,13 |
| murA | target alteration | fosfomycin | 0 | 0 | 21 | 4 | 0,11 |
| RbpA | target alteration | rifampin | 0 | 0 | 22 | 2 | 0,11 |
| mexi | efflux pump | multidrug | 0 | 0 | 18 | 5 | 0,1 |
| mepA | efflux pump | multidrug | 0 | 0 | 13 | 7 | 0,09 |
| RosA | efflux pump | fosmidomycin | 0 | 0 | 4 | 14 | 0,08 |
| ttgC | efflux pump | multidrug | 0 | 0 | 13 | 3 | 0,07 |
| APH(6)-Id | inactivation | aminoglycoside | 0 | 0 | 5 | 10 | 0,07 |
| arnC | target alteration | polymyxin | 0 | 0 | 5 | 5 | 0,04 |
| arnD | target alteration | glycopeptide | 0 | 0 | 6 | 2 | 0,04 |
| MexD | efflux pump | multidrug | 0 | 0 | 3 | 5 | 0,04 |
| pbp2 | target replacement | beta_lactam | 0 | 0 | 4 | 3 | 0,03 |

* ARGs are listed in descending order according to their total abundance in contigs of all apple samples.
